# 2-(2,3-Dimethyl­phen­yl)-1*H*-isoindole-1,3(2*H*)-dione

**DOI:** 10.1107/S1600536810033386

**Published:** 2010-08-28

**Authors:** Muhammad Ilyas Tariq, Tanzeela Noreen, M. Nawaz Tahir, Shahbaz Ahmad, Muhammad Fayyaz-ur-Rehman

**Affiliations:** a Department of Chemistry, University of Sargodha, Sargodha, Pakistan; b Department of Physics, University of Sargodha, Sargodha, Pakistan

## Abstract

In the title compound, C_16_H_13_NO_2_, the 2,3-dimethyl­phenyl group and the 1*H*-isoindole-1,3(2*H*)-dione group are essentially planar, with r.m.s. deviations of 0.006 and 0.013 Å, respectively, and are oriented at an angle of 78.19 (3)° with respect to each other. In the crystal, weak C—H⋯O inter­actions link the mol­ecules, forming a zigzag chain parallel to the *b* axis. Futhermore, C—H⋯π inter­actions are present between the C—H group of isoindole and the 2,3-dimethyl­phenyl benzene ring. The H atoms of the *ortho*-methyl group are statistically disordered over two positions. Such disorder might be related to the antagonism between intra­molecular steric repulsions and inter­molecular C—H⋯O inter­actions.

## Related literature

For background to Schiff bases containing 2,3-dimethyl­aniline and for related structures, see: Bocelli & Cantoni (1989 ▶); Chandrashekar *et al.* (1983 ▶); Izotova *et al.* (2009 ▶); Sarfraz *et al.* (2010 ▶); Tahir *et al.* (2010 ▶); Tariq *et al.* (2010 ▶).
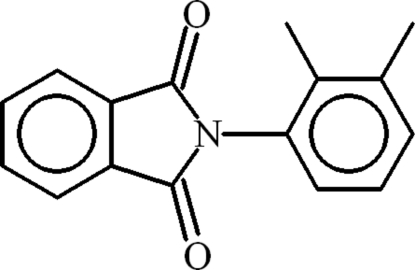


## Experimental

### 

#### Crystal data

C_16_H_13_NO_2_
*M* *_r_* = 251.27
Monoclinic, 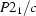

*a* = 7.8222 (3) Å
*b* = 8.4576 (3) Å
*c* = 19.4863 (6) Å
β = 91.441 (2)°
*V* = 1288.75 (8) Å^3^
*Z* = 4
Mo *K*α radiation
μ = 0.09 mm^−1^
*T* = 296 K
0.30 × 0.12 × 0.10 mm

#### Data collection

Bruker APEXII CCD diffractometer
Absorption correction: multi-scan (*SADABS*; Bruker, 2005 ▶) *T*_min_ = 0.980, *T*_max_ = 0.993
9849 measured reflections
2320 independent reflections
1819 reflections with *I* > 2σ(*I*)
*R*_int_ = 0.029

#### Refinement

*R*[*F* ^2^ > 2σ(*F* ^2^)] = 0.038
*wR*(*F* ^2^) = 0.097
*S* = 1.04
2320 reflections
173 parameters
H-atom parameters constrained
Δρ_max_ = 0.13 e Å^−3^
Δρ_min_ = −0.13 e Å^−3^

### 

Data collection: *APEX2* (Bruker, 2009 ▶); cell refinement: *SAINT* (Bruker, 2009 ▶); data reduction: *SAINT*; program(s) used to solve structure: *SHELXS97* (Sheldrick, 2008 ▶); program(s) used to refine structure: *SHELXL97* (Sheldrick, 2008 ▶); molecular graphics: *ORTEP-3 for Windows* (Farrugia, 1997 ▶) and *PLATON* (Spek, 2009 ▶); software used to prepare material for publication: *WinGX* (Farrugia, 1999 ▶) and *PLATON*.

## Supplementary Material

Crystal structure: contains datablocks global, I. DOI: 10.1107/S1600536810033386/dn2596sup1.cif

Structure factors: contains datablocks I. DOI: 10.1107/S1600536810033386/dn2596Isup2.hkl

Additional supplementary materials:  crystallographic information; 3D view; checkCIF report

## Figures and Tables

**Table 1:** Hydrogen-bond geometry (Å, °) *Cg*2 is the centroid of the C1—C6 ring.

*D*—H⋯*A*	*D*—H	H⋯*A*	*D*⋯*A*	*D*—H⋯*A*
C7—H7*F*⋯O1^i^	0.96	2.52	3.3486 (19)	145
C8—H8*C*⋯*Cg*2^ii^	0.96	2.89	3.5644 (18)	128
C11—H11⋯*Cg*2^iii^	0.93	2.77	3.6798 (15)	166

Symmetry codes: (i) 
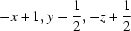
; (ii) 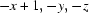; (iii) 
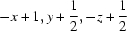
.

**Fig. 1.**
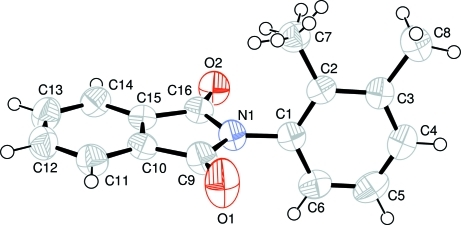
View of the title compound with the atom numbering scheme. The thermal ellipsoids are drawn at the 50% probability level. H-atoms are represented by small circles of arbitrary radii.

## supplementary crystallographic information

### Comment

We have reported crystal structures of Schiff bases containing 2,3-dimethylaniline (Sarfraz *et al.*, 2010), (Tahir *et al.*, 2010) and (Tariq *et al.*, 2010). As part of our continuing interest in Schiff bases containing 2,3-dimethylaniline, we report here the structure of the title compound (I).

The crystal structure of related compounds (II), *N*-phenylphthalimide (Izotova *et al.*, 2009), (III) *N*-*m*-tolylphthalimide (Chandrashekar *et al.*, 1983) and (IV) *N*-(*o*-tolyl)phthalimide (Bocelli & Cantoni, 1989) have been already published.

In (I), the 2,3-dimethylanilinic moiety A (C1—C8) and the 1*H*-isoindole-1,3(2*H*)-dione group B (C9—C15/N1/O1/O2) are planar with r. m. s. deviations of 0.0056 and 0.0131 Å, respectively. The dihedral angle between A/B is 78.19 (3)° (Fig. 1).

The H-atoms of the *ortho*-methyl group are statistically disordered over two positions. Such disorder might be related to the antagonism between intramolecular C-H···N and intermolecular C-H···O interactions (Table 1). The weak C—H···O interactions links the molecule forming a non continuous zig-zag chain parallel to the b axis owing to the statistical distribution of the H atoms. Weak C-H···π interactions are also present (Table 1, where Cg2 is the centroid of the phenyl ring C1—C6).

### Experimental

Equimolar quantities of 2,3-dimethylaniline and phthalic anhydride were refluxed in methanol for 48 h. The solution was kept at room temperature which affoarded white prism after 48 h.

### Refinement

The H-atoms were positioned geometrically (C–H = 0.93–0.96 Å) and refined as riding with *U*_iso_(H) = *xU*_eq_(C), where *x* = 1.5 for methyl and *x* = 1.2 for aryl H-atoms.

The difference Fourier map showed that H-atoms of *ortho*-methyl are disordered. They were then geometrically located and treated as riding using the tools (AFIX 123) available in SHELXL97 (Sheldrick, 2008)

### Crystal data

**Table:**

C_16_H_13_NO_2_	*F*(000) = 528
*M**_r_* = 251.27	*D*_x_ = 1.295 Mg m^−^^3^
Monoclinic, *P*2_1_/*c*	Mo *K*α radiation, λ = 0.71073 Å
Hall symbol: -P 2ybc	Cell parameters from 1819 reflections
*a* = 7.8222 (3) Å	θ = 2.6–25.2°
*b* = 8.4576 (3) Å	µ = 0.09 mm^−^^1^
*c* = 19.4863 (6) Å	*T* = 296 K
β = 91.441 (2)°	Prism, white
*V* = 1288.75 (8) Å^3^	0.30 × 0.12 × 0.10 mm
*Z* = 4	

### Data collection

**Table:**

Bruker APEXII CCD diffractometer	2320 independent reflections
Radiation source: fine-focus sealed tube	1819 reflections with *I* > 2σ(*I*)
graphite	*R*_int_ = 0.029
Detector resolution: 8.10 pixels mm^-1^	θ_max_ = 25.2°, θ_min_ = 2.6°
ω scans	*h* = −8→9
Absorption correction: multi-scan (*SADABS*; Bruker, 2005)	*k* = −10→10
*T*_min_ = 0.980, *T*_max_ = 0.993	*l* = −23→23
9849 measured reflections	

### Refinement

**Table:**

Refinement on *F*^2^	Primary atom site location: structure-invariant direct methods
Least-squares matrix: full	Secondary atom site location: difference Fourier map
*R*[*F*^2^ > 2σ(*F*^2^)] = 0.038	Hydrogen site location: inferred from neighbouring sites
*wR*(*F*^2^) = 0.097	H-atom parameters constrained
*S* = 1.04	*w* = 1/[σ^2^(*F*_o_^2^) + (0.0408*P*)^2^ + 0.2276*P*] where *P* = (*F*_o_^2^ + 2*F*_c_^2^)/3
2320 reflections	(Δ/σ)_max_ < 0.001
173 parameters	Δρ_max_ = 0.13 e Å^−^^3^
0 restraints	Δρ_min_ = −0.13 e Å^−^^3^

### Special details

**Table:**

Geometry. All esds (except the esd in the dihedral angle between two l.s. planes) are estimated using the full covariance matrix. The cell esds are taken into account individually in the estimation of esds in distances, angles and torsion angles; correlations between esds in cell parameters are only used when they are defined by crystal symmetry. An approximate (isotropic) treatment of cell esds is used for estimating esds involving l.s. planes.
Refinement. Refinement of *F*^2^ against ALL reflections. The weighted *R*-factor *wR* and goodness of fit *S* are based on *F*^2^, conventional *R*-factors *R* are based on *F*, with *F* set to zero for negative *F*^2^. The threshold expression of *F*^2^ > σ(*F*^2^) is used only for calculating *R*-factors(gt) *etc*. and is not relevant to the choice of reflections for refinement. *R*-factors based on *F*^2^ are statistically about twice as large as those based on *F*, and *R*- factors based on ALL data will be even larger.

### Fractional atomic coordinates and isotropic or equivalent isotropic displacement parameters (Å^2^)

**Table:**

	*x*	*y*	*z*	*U*_iso_*/*U*_eq_	Occ. (<1)
O1	0.49485 (18)	0.42949 (17)	0.22907 (6)	0.0925 (5)	
O2	0.79930 (14)	0.38827 (14)	0.03357 (5)	0.0628 (3)	
N1	0.61379 (16)	0.38436 (14)	0.12397 (6)	0.0490 (3)	
C1	0.49387 (19)	0.26593 (18)	0.10015 (7)	0.0467 (4)	
C2	0.53838 (18)	0.10792 (18)	0.10503 (6)	0.0450 (4)	
C3	0.41581 (19)	−0.00524 (18)	0.08357 (7)	0.0489 (4)	
C4	0.2583 (2)	0.0467 (2)	0.05916 (7)	0.0598 (4)	
H4	0.1775	−0.0278	0.0449	0.072*	
C5	0.2167 (2)	0.2044 (2)	0.05521 (8)	0.0671 (5)	
H5	0.1092	0.2354	0.0388	0.080*	
C6	0.3357 (2)	0.3165 (2)	0.07580 (8)	0.0579 (4)	
H6	0.3099	0.4237	0.0733	0.069*	
C7	0.7094 (2)	0.0574 (2)	0.13251 (8)	0.0591 (4)	
H7A	0.7165	−0.0559	0.1318	0.089*	0.50
H7B	0.7247	0.0945	0.1788	0.089*	0.50
H7C	0.7970	0.1010	0.1046	0.089*	0.50
H7D	0.7756	0.1490	0.1450	0.089*	0.50
H7E	0.7675	−0.0015	0.0980	0.089*	0.50
H7F	0.6951	−0.0079	0.1723	0.089*	0.50
C8	0.4548 (2)	−0.1781 (2)	0.08813 (9)	0.0665 (5)	
H8A	0.3620	−0.2372	0.0677	0.100*	
H8B	0.4697	−0.2079	0.1354	0.100*	
H8C	0.5579	−0.2001	0.0642	0.100*	
C9	0.6040 (2)	0.45618 (19)	0.18862 (7)	0.0555 (4)	
C10	0.75207 (18)	0.56363 (17)	0.19457 (7)	0.0457 (4)	
C11	0.8017 (2)	0.66315 (19)	0.24722 (7)	0.0562 (4)	
H11	0.7393	0.6707	0.2871	0.067*	
C12	0.9479 (2)	0.7513 (2)	0.23830 (8)	0.0622 (4)	
H12	0.9848	0.8199	0.2729	0.075*	
C13	1.0403 (2)	0.7399 (2)	0.17923 (9)	0.0652 (5)	
H13	1.1387	0.8005	0.1749	0.078*	
C14	0.9898 (2)	0.6399 (2)	0.12609 (8)	0.0565 (4)	
H14	1.0520	0.6326	0.0862	0.068*	
C15	0.84406 (18)	0.55212 (16)	0.13486 (7)	0.0432 (3)	
C16	0.75805 (19)	0.43515 (17)	0.08932 (7)	0.0463 (4)	

### Atomic displacement parameters (Å^2^)

**Table:**

	*U*^11^	*U*^22^	*U*^33^	*U*^12^	*U*^13^	*U*^23^
O1	0.0969 (10)	0.1151 (11)	0.0678 (8)	−0.0452 (9)	0.0465 (8)	−0.0311 (7)
O2	0.0700 (8)	0.0772 (8)	0.0420 (6)	−0.0077 (6)	0.0174 (5)	−0.0097 (5)
N1	0.0550 (8)	0.0515 (7)	0.0412 (6)	−0.0075 (6)	0.0130 (5)	−0.0041 (5)
C1	0.0521 (9)	0.0521 (9)	0.0363 (7)	−0.0015 (7)	0.0086 (6)	−0.0002 (6)
C2	0.0462 (8)	0.0558 (9)	0.0332 (7)	0.0017 (7)	0.0064 (6)	0.0019 (6)
C3	0.0528 (9)	0.0580 (9)	0.0364 (7)	−0.0068 (7)	0.0081 (6)	0.0004 (6)
C4	0.0569 (10)	0.0754 (12)	0.0469 (9)	−0.0121 (9)	0.0012 (7)	0.0016 (8)
C5	0.0491 (10)	0.0938 (14)	0.0580 (10)	0.0064 (10)	−0.0028 (8)	0.0117 (9)
C6	0.0560 (10)	0.0646 (10)	0.0532 (9)	0.0073 (8)	0.0041 (7)	0.0079 (8)
C7	0.0575 (10)	0.0606 (10)	0.0591 (9)	0.0025 (8)	0.0014 (8)	0.0047 (8)
C8	0.0784 (13)	0.0584 (10)	0.0630 (10)	−0.0083 (9)	0.0117 (9)	−0.0041 (8)
C9	0.0642 (10)	0.0581 (10)	0.0450 (8)	−0.0058 (8)	0.0175 (7)	−0.0056 (7)
C10	0.0514 (9)	0.0451 (8)	0.0410 (7)	0.0039 (7)	0.0063 (6)	0.0009 (6)
C11	0.0627 (11)	0.0614 (10)	0.0450 (8)	0.0008 (8)	0.0077 (7)	−0.0068 (7)
C12	0.0637 (11)	0.0645 (11)	0.0581 (10)	−0.0053 (9)	−0.0037 (8)	−0.0118 (8)
C13	0.0554 (10)	0.0726 (12)	0.0677 (11)	−0.0122 (9)	0.0030 (8)	−0.0031 (9)
C14	0.0520 (10)	0.0662 (10)	0.0516 (9)	−0.0021 (8)	0.0103 (7)	0.0020 (8)
C15	0.0460 (8)	0.0444 (8)	0.0394 (7)	0.0049 (7)	0.0045 (6)	0.0039 (6)
C16	0.0507 (9)	0.0494 (9)	0.0392 (7)	0.0038 (7)	0.0090 (6)	0.0029 (6)

### Geometric parameters (Å, °)

**Table:**

O1—C9	1.1982 (17)	C7—H7D	0.9600
O2—C16	1.2079 (16)	C7—H7E	0.9600
N1—C16	1.3972 (17)	C7—H7F	0.9600
N1—C9	1.4025 (18)	C8—H8A	0.9600
N1—C1	1.4411 (19)	C8—H8B	0.9600
C1—C6	1.382 (2)	C8—H8C	0.9600
C1—C2	1.384 (2)	C9—C10	1.474 (2)
C2—C3	1.411 (2)	C10—C11	1.375 (2)
C2—C7	1.491 (2)	C10—C15	1.3867 (18)
C3—C4	1.381 (2)	C11—C12	1.380 (2)
C3—C8	1.496 (2)	C11—H11	0.9300
C4—C5	1.375 (3)	C12—C13	1.378 (2)
C4—H4	0.9300	C12—H12	0.9300
C5—C6	1.381 (2)	C13—C14	1.386 (2)
C5—H5	0.9300	C13—H13	0.9300
C6—H6	0.9300	C14—C15	1.375 (2)
C7—H7A	0.9600	C14—H14	0.9300
C7—H7B	0.9600	C15—C16	1.479 (2)
C7—H7C	0.9600		
			
C16—N1—C9	111.33 (12)	C2—C7—H7F	109.5
C16—N1—C1	125.81 (11)	H7A—C7—H7F	56.3
C9—N1—C1	122.73 (11)	H7B—C7—H7F	56.3
C6—C1—C2	122.96 (14)	H7C—C7—H7F	141.1
C6—C1—N1	117.74 (14)	H7D—C7—H7F	109.5
C2—C1—N1	119.25 (13)	H7E—C7—H7F	109.5
C1—C2—C3	117.84 (14)	C3—C8—H8A	109.5
C1—C2—C7	121.57 (14)	C3—C8—H8B	109.5
C3—C2—C7	120.59 (14)	H8A—C8—H8B	109.5
C4—C3—C2	118.69 (15)	C3—C8—H8C	109.5
C4—C3—C8	120.68 (15)	H8A—C8—H8C	109.5
C2—C3—C8	120.62 (15)	H8B—C8—H8C	109.5
C5—C4—C3	122.39 (16)	O1—C9—N1	124.41 (15)
C5—C4—H4	118.8	O1—C9—C10	129.49 (14)
C3—C4—H4	118.8	N1—C9—C10	106.09 (11)
C4—C5—C6	119.53 (16)	C11—C10—C15	121.76 (14)
C4—C5—H5	120.2	C11—C10—C9	129.95 (13)
C6—C5—H5	120.2	C15—C10—C9	108.29 (12)
C5—C6—C1	118.59 (16)	C10—C11—C12	117.11 (14)
C5—C6—H6	120.7	C10—C11—H11	121.4
C1—C6—H6	120.7	C12—C11—H11	121.4
C2—C7—H7A	109.5	C13—C12—C11	121.42 (15)
C2—C7—H7B	109.5	C13—C12—H12	119.3
H7A—C7—H7B	109.5	C11—C12—H12	119.3
C2—C7—H7C	109.5	C12—C13—C14	121.41 (16)
H7A—C7—H7C	109.5	C12—C13—H13	119.3
H7B—C7—H7C	109.5	C14—C13—H13	119.3
C2—C7—H7D	109.5	C15—C14—C13	117.26 (14)
H7A—C7—H7D	141.1	C15—C14—H14	121.4
H7B—C7—H7D	56.3	C13—C14—H14	121.4
H7C—C7—H7D	56.3	C14—C15—C10	121.05 (13)
C2—C7—H7E	109.5	C14—C15—C16	130.74 (12)
H7A—C7—H7E	56.3	C10—C15—C16	108.21 (12)
H7B—C7—H7E	141.1	O2—C16—N1	124.82 (14)
H7C—C7—H7E	56.3	O2—C16—C15	129.14 (13)
H7D—C7—H7E	109.5	N1—C16—C15	106.04 (11)

### Hydrogen-bond geometry (Å, °)

**Table:**

Cg2 is the centroid of the C1—C6 ring.

**Table:**

*D*—H···*A*	*D*—H	H···*A*	*D*···*A*	*D*—H···*A*
C7—H7D···N1	0.96	2.39	2.868 (2)	110.
C7—H7F···O1^i^	0.96	2.52	3.3486 (19)	145.
C8—H8C···Cg2^ii^	0.96	2.89	3.5644 (18)	128
C11—H11···Cg2^iii^	0.93	2.77	3.6798 (15)	166

Symmetry codes: (i) −*x*+1, *y*−1/2, −*z*+1/2; (ii) −*x*+1, −*y*, −*z*; (iii) −*x*+1, *y*+1/2, −*z*+1/2.
